# Rebalancing β-Amyloid-Induced Decrease of ATP Level by Amorphous Nano/Micro Polyphosphate: Suppression of the Neurotoxic Effect of Amyloid β-Protein Fragment 25-35

**DOI:** 10.3390/ijms18102154

**Published:** 2017-10-16

**Authors:** Werner E. G. Müller, Shunfeng Wang, Maximilian Ackermann, Meik Neufurth, Renate Steffen, Egherta Mecja, Rafael Muñoz-Espí, Qingling Feng, Heinz C. Schröder, Xiaohong Wang

**Affiliations:** 1 ERC Advanced Investigator Grant Research Group at the Institute for Physiological Chemistry, University Medical Center of the Johannes Gutenberg University, Duesbergweg 6, D-55128 Mainz, Germany; shunwang@uni-mainz.de (S.W.); mneufurt@uni-mainz.de (M.N.); steffen@uni-mainz.de (R.S.); egherta@gmail.com (E.M.); hschroed@uni-mainz.de (H.C.S.); 2 Institute of Functional and Clinical Anatomy, University Medical Center of the Johannes Gutenberg University, Johann Joachim Becher Weg 13, D-55099 Mainz, Germany; maximilian.ackermann@uni-mainz.de; 3 Institute of Materials Science (ICMUV), Universitat de València, C/Catedràtic José Beltrán 2, 46980 Paterna, València, Spain; rafael.munoz@uv.es; 4 Key Laboratory of Advanced Materials of Ministry of Education of China, School of Materials Science and Engineering, Tsinghua University, Beijing 100084, China; biomater@mail.tsinghua.edu.cn

**Keywords:** β-amyloid, calcium polyphosphate, microparticles, neurotoxic effect, adenosine triphosphate level, PC12 cells, primary rat cortex neurons

## Abstract

Morbus Alzheimer neuropathology is characterized by an impaired energy homeostasis of brain tissue. We present an approach towards a potential therapy of Alzheimer disease based on the high-energy polymer inorganic polyphosphate (polyP), which physiologically occurs both in the extracellular and in the intracellular space. Rat pheochromocytoma (PC) 12 cells, as well as rat primary cortical neurons were exposed to the Alzheimer peptide Aβ25-35. They were incubated in vitro with polyphosphate (polyP); ortho-phosphate was used as a control. The polymer remained as Na^+^ salt; or complexed in a stoichiometric ratio to Ca^2+^ (Na-polyP[Ca^2+^]); or was processed as amorphous Ca-polyP microparticles (Ca-polyP-MP). Ortho-phosphate was fabricated as crystalline Ca-phosphate nanoparticles (Ca-phosphate-NP). We show that the pre-incubation of PC12 cells and primary cortical neurons with polyP protects the cells against the neurotoxic effect of the Alzheimer peptide Aβ25-35. The strongest effect was observed with amorphous polyP microparticles (Ca-polyP-MP). The effect of the soluble sodium salt; Na-polyP (Na-polyP[Ca^2+^]) was lower; while crystalline orthophosphate nanoparticles (Ca-phosphate-NP) were ineffective. Ca-polyP-MP microparticles and Na-polyP[Ca^2+^] were found to markedly enhance the intracellular ATP level. Pre-incubation of Aβ25-35 during aggregate formation, with the polyP preparation before exposure of the cells, had a small effect on neurotoxicity. We conclude that recovery of the compromised energy status in neuronal cells by administration of nontoxic biodegradable Ca-salts of polyP reverse the β-amyloid-induced decrease of adenosine triphosphate (ATP) level. This study contributes to a new routes for a potential therapeutic intervention in Alzheimer’s disease pathophysiology.

## 1. Introduction

Alzheimer’s disease (AD), a neurodegenerative disorder characterized by deposition of amyloid-β (Aβ) peptides in the brain, leads to progressive memory loss and dementia [1,2]. The two proteins, amyloid β-protein and Tau, are characteristic signatures of AD [3]. Amyloid β-protein is a small peptide fragment generated through cleavage from the amyloid precursor protein (APP) [4]. Due to the fact that the etiologic agent(s) and the metabolic targets for AD have not been identified unequivocally, no causative cure can be offered. Five agents have been accepted with sufficient safety and efficacy to allow marketing approval: four cholinesterase inhibitors [5] and memantine [6]. We could demonstrate that memantine prevents neuronal apoptosis due to modulation of the NMDA (*N*-methyl-d-aspartate) receptor [7,8].

In general neurons and glial cells consume high levels of ATP in the brain. Since no energy storage system is available in the central nervous system, there is a high demand for a continuous adenosine triphosphate (ATP) supply that must be guaranteed to maintain energy homeostasis [9,10]. In our previous studies, we reported that the level of inorganic polyphosphate (polyP) in the brain of rats decrease with age [11,12]. Especially the latter finding is interesting since it might imply that the reduced polyP concentration in the brain of older specimens (rats) might have an etiological role or represent a predisposition for Alzheimer’s disease. polyP, consisting of up to 1000 phosphoanhydride bond-linked phosphate monomers, has been detected in all prokaryotic and eukaryotic organisms; the concentration in mammalian tissues is approximately 50 μM, equivalent to 5 mg/g of tissue [13]. Intracellularly, polyP is present in the cytosol and in 100–200 nm sized acidocalcisomes, and is secreted into the extracellular space by platelets, astrocytes, and bacteria [14]. During hydrolysis of polyP by the enzyme alkaline phosphatase energy-rich anhydride bonds are cleaved that are proposed to give rise to metabolic energy ADP and/or ATP [15,16].

Recently, it has been published that polyP accelerates the fibril formation of both bacterial and human proteins [16,17]. Furthermore, experiments showed that the intracellular level of polyP might confer to a protection against cell death, including the β-amyloid peptide induced Ca^2+^-dependent apoptosis [18]. Now, we used rat pheochromocytoma cells [PC12 cells], as well as primary cortical neurons, to study the effect of the soluble Na^+^ salt of polyP (Na-polyP) and of amorphous microparticles fabricated from the Ca^2+^ salt of polyP (Ca-polyP-MP) to determine the neuroprotective potency of these two polymer preparations. As an inducer of amyloid β-protein-related cell toxicity, we applied a fragment of the amyloid β-protein, displaying amyloidogenic ability, primarily Aβ25-35 [19,20]. In a previous study, we reported that the Aβ peptide should stay in distilled water in a stock solution (900 μM) for five days (5 day) to develop full toxicity [21,22]. In the present study, we added Aβ25-35 at the clinical relevant dose of 5 μM (see: [23]) to PC12 cells. This Aβ25-35 peptide was found to reduce the cell survival during a 6 h incubation period. Concurrently, the ATP level of the cells dropped in the presence of Aβ25-35 by more than 50%. Both this toxic effect and the drop of the ATP pool size could be partially reversed by Na-polyP and almost completely by Ca-polyP-MP. We conclude that polyP rebalances of the β-amyloid-induced decrease in ATP level and in turn abrogate the neurotoxic effect displayed by Aβ25-35.

## 2. Results

### 2.1. Fabrication and Morphology of the Particles

In previous studies, it had been suspected that polyP microparticles are taken up by endocytosis [15,16], while the soluble polyP interacts with cell-surface receptors, followed by a signal-transduction pathway [24]. Therefore, the studies, summarized here had to be performed in parallel with both soluble (Na-polyP[Ca^2+^]) and particulate (Ca-phosphate-NP, Ca-polyP-MP) phosphate samples.

The particles were fabricated using a previously developed precipitation method from CaCl_2_ and an aqueous polyphosphate solution at an approximate weight ratio of 2:1 [15]. Under these weight conditions, polyP was found to form microspheres. This procedure was applied both for Na-polyP, with a chain length of ~40 P*_i_* units, and tri-sodium (ortho)-phosphate. The CaCl_2_ solution was added dropwise to the respective phosphate solution. Na-polyP[Ca^2+^] was prepared as described under “Materials and Methods”.

**Figure 1:**
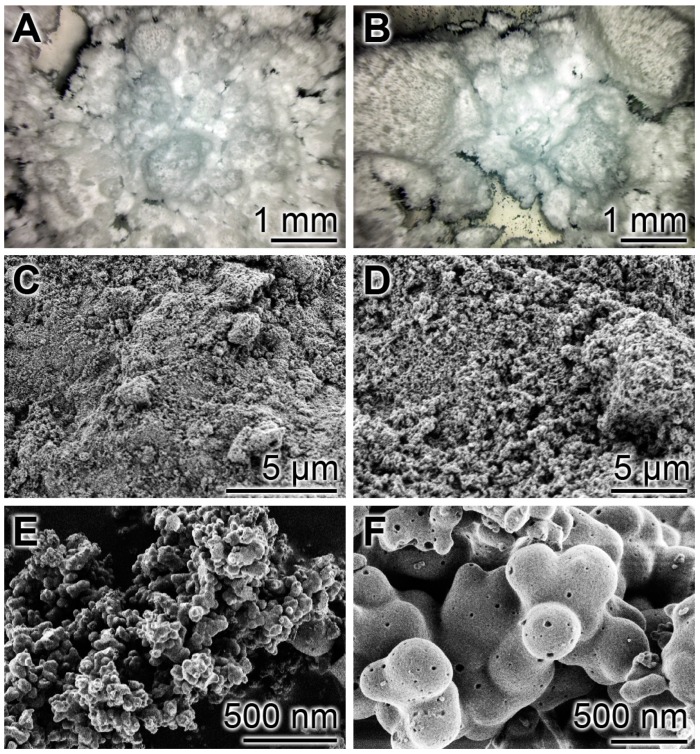
Micrographs of Ca-phosphate nanoparticles (Ca-phosphate-NP) and of Ca-polyP microparticles (Ca-polyP-MP); (**A**,**B**) optical microscopy; (**C**–**F**) Scanning electron microscopy (SEM) (**A**,**C**,**D**) The Ca-phosphate-NP appear as homogeneous material and as spherical particles of a size around 35 nm at high magnification; (**B**,**E**,**F**) The Ca-polyP-MP particles are a likewise homogenous powder at lower magnification and spherical particles at high power scanning electron microscopy (SEM).

The fabricated particles, both Ca-phosphate-NP and Ca-polyP-MP, had a powder like consistency (Figure 1A,B). At a higher magnification they appear as homogeneous grains (Figure 1C,E). At the nanoscale, the Ca-phosphate-NP show a largely homogeneous morphology with a diameter of the particles of 35 ± 8 nm (*n* = 20) (Figure 1D). In contrast, the spherical Ca-polyP-MP showed an average size of 170 ± 87 nm (Figure 1F).

### 2.2. Characterization by Fourier Transform Infrared and X-ray Diffraction

**Figure 2:**
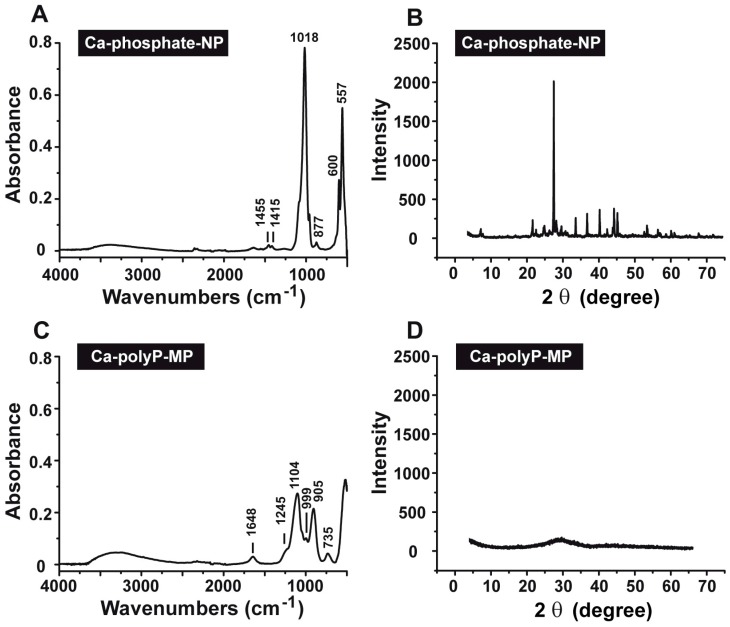
Characterization of the (**A**,**B**) Ca-phosphate-NP and (**C**,**D**) Ca-polyP-MP particles. The analyses were performed by (**A**,**C**) Fourier Transform Infrared (FTIR) and (**B**,**D**) X-Ray Diffraction (XRD).

The Fourier Transform Infrared (FTIR) spectra of the Ca-phosphate-NP (Figure 2A) and the Ca-polyP-MP (Figure 2C) show characteristic differences. The Ca-phosphate-NP exhibit a spectrum indicative for carbonated apatite [25]. The spectrum comprises the typical ν_4_ bending vibrations of PO_4_^3−^ at 557 and 600 cm^−1^, the ν_1_ symmetric PO_4_^3−^ stretching at 960 cm^−1^ (to be published), as well as the ν_3_ asymmetric stretching at 1018 cm^−1^. The occurrence of the latter band is also proven to be a marker for ortho-phosphate [26]. Additionally, bands originating from carbonate are visible at 877 cm^−1^ (ν_2_ bending vibration) and 1415 cm^−1^ as well as 1455 cm^−1^ (ν_3_ asymmetric stretching vibration; double band). The occurrence of these CO_3_^2−^ bands is characteristic for type B apatite [27,28]. In contrast, the spectrum of the Ca-polyP-MP (Figure 2C) only shows the characteristic signals for polyP, as described before [15]. These can be ascribed with 1245 cm^−1^ for ν_as_ (PO_2_)^−^, 1104 cm^−1^ for ν_as_ (PO_3_)^2−^, 997 cm^−1^ for ν_sym_ (PO_3_)^2−^, 905 cm^−1^ for ν_as_ (P-O-P) and 735 cm^−1^ for ν_sym_ (P–O–P). Vibrations indicative for carbonate are not present.

The X-Ray Diffraction (XRD) pattern for Ca-phosphate-NP shows that the mineral is crystalline (Figure 2B). This must be deduced from the recorded pattern between 20° and 57°; there, sharp reflections are seen with the maximum peak at 26.4°. In contrast, the XRD pattern for Ca-polyP-MP indicates that this material is amorphous (Figure 2D).

### 2.3. Cell viability after Exposure to Phosphate or polyP Preparations

**Figure 3:**
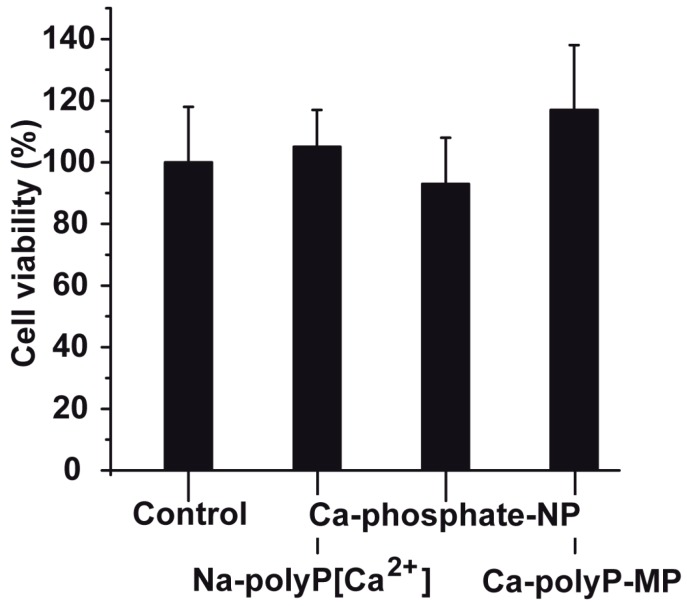
Viability of PC12 cells seeded into 48-well plates for a period of 72 h. Cell viability was determined using the MTT (3-[4,5-dimethyl thiazole-2-yl]-2,5-diphenyl tetrazolium) assay. The concentrations of Na-polyP[Ca^2+^], Ca-phosphate-NP and Ca-polyP-MP in the assays were identical (30 μg/mL); the controls did not contain additional phosphate or polyP. Data represent mean ± SD of ten independent experiments; no significant differences are calculated (*p* > 0.05).

PC12 cells were exposed to three different phosphate preparations (concentration 30 μg/mL), first against Na-polyP[Ca^2+^], then against Ca-phosphate-NP, and finally against Ca-polyP-MP (Figure 3). In the controls, no phosphate sample was added. The incubation in the 48-well plates was for 72 h; the seeding concentration was 2 × 10^4^ cells/mL. At the end of the incubation period, the cells were harvested and subjected to the 3-[4,5-dimethyl thiazole-2-yl]-2,5-diphenyl tetrazolium (MTT) assay; the amount of formazan crystals was quantitated as described under “Materials and Methods”. As seen the viability, measured on the basis of the enzymatic reaction, was found to be for all three preparations not significantly different if compared to the control (without phosphate or polyP); Figure 3. In more detailed viability tests the different phosphate and polyP samples were tested in the concentration range of 3 to 100 μg/mL. In none of the assays the viability in the controls was significantly different than those in the test series (to be published).

### 2.4. Induced Toxicity by the Amyloid β-Protein Peptide (Time-Dependent Pre-Incubation of Aβ25-35 in Water)

**Figure 4:**
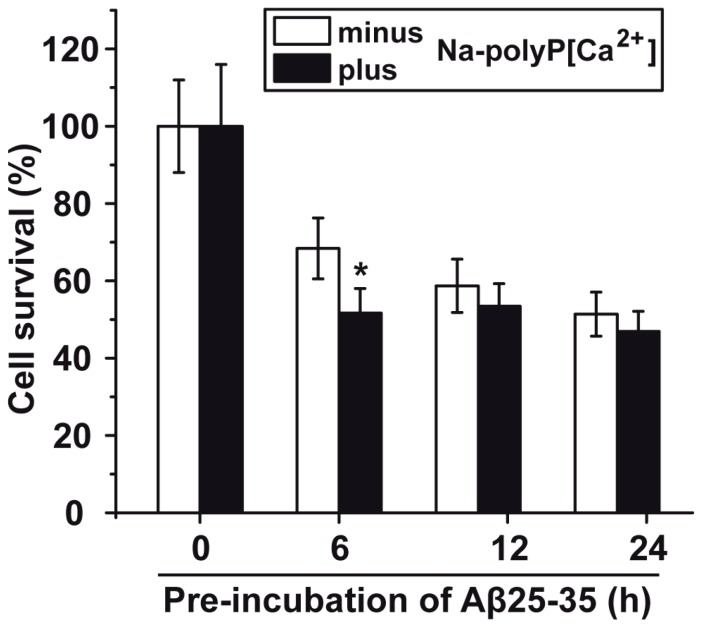
Viability of PC12 cells incubated with the amyloid β-protein fragment Aβ25-35 that had been pre-incubated with Na-polyP[Ca^2+^]. The peptide had been pre-incubated in distilled water for 6 to 24 h, or was immediately used after solubilization. During the pre-incubation period Aβ25-35 remained untreated or was treated with 30 μg/mL of Na-polyP[Ca^2+^] (minus/plus Na-polyP[Ca^2+^]), as described under “Materials and Methods”. After that, the respective peptide sample was added to the cell culture (8 × 10^4^ PC12 cells/mL) at a concentration of 5 μM and incubated for 12 h. Then the viability of the cells was determined by the MTT assay; the cell survival rate was calculated and is given as % to the corresponding control culture. Data (±SD) have been based on ten independent experiments; (*) the significance has been calculated (*p* < 0.05).

In order to assess at which conditions Aβ25-35 should be used for the toxicity/protection assays using PC12 cells one series of experiments were performed with Na-polyP[Ca^2+^] and Aβ25-35. The peptide had been pre-incubated for a different period of time (0 to 24 h). The Aβ25-35 peptide was dissolved in distilled water (900 μM). Then, the peptide was added either immediately to the cells or was pre-incubated for 6 to 24 h in water and then added to the PC12 cells; the final concentration of the Aβ25-35 peptide was 5 μM. The concentration of cells was adjusted to 8 × 10^4^ cells/mL. Subsequently, the cells were incubated for 12 h followed by the determination of the viability, using the MTT assay system. The results (Figure 4) show that the Aβ25-35 peptide, pre-incubated for 6 h or longer, caused a significant reduction of the PC12 cell number (measured on the basis of the viability of the cells in the assay), by approximately 50–60%. In order to assess the toxic effect of Aβ25-35, in dependence of the period of pre-incubation, the PC12 cells were exposed to Aβ25-35 peptide, which had been pre-incubated with 30 μg/mL of Na-polyP[Ca^2+^] for 0 to 24 h. In this series only the values for the 6 h pre-incubation period showed a significant difference between the polyP-untreated and polyP-pretreated fragment. The results show that the Aβ25-35, fragment pre-incubated with polyP, is about 15% more toxic (Figure 4). At the present state of knowledge, we attribute this increase in the toxicity to a transient stabilization/conformational change due to the binding of polyP to the peptide of the amyloid β-protein, as outlined before [17]. This intensifying effect (toxicity) of Na-polyP[Ca^2+^] on the cell growth/viability could not been detected with Ca-phosphate-NP, or Ca-polyP-MP, under otherwise identical conditions.

In a preliminary series of experiments we did not find any change of the degree of toxicity of amyloid β-protein after incubation of the peptide with polyP nanoparticles. Aβ25-35 had been incubated with the nanoparticles for 30 min followed by a centrifugation step (100,000 *g*/30 min); then the supernatant was tested for toxicity (to be published).

### 2.5. Protection against Aβ25-35-Caused Toxicity by polyP

Based on the preceding results, we determined the (potential) cytoprotective effect of polyP, by using an Aβ25-35 sample that had been pre-incubated for 6 h in aqueous solution. In parallel, the PC12 cells were pre-incubated with 30 μg/mL of the phosphate/polyP samples (either Na-polyP[Ca^2+^], Ca-phosphate-NP, or Ca-polyP-MP) for 24 h. Then, the phosphate-pre-incubated cells assays were mixed with Aβ25-35 (5 μM Aβ25-35 (final concentration)). In the controls to these experiments, the cells were only exposed to the toxic peptide.

**Figure 5:**
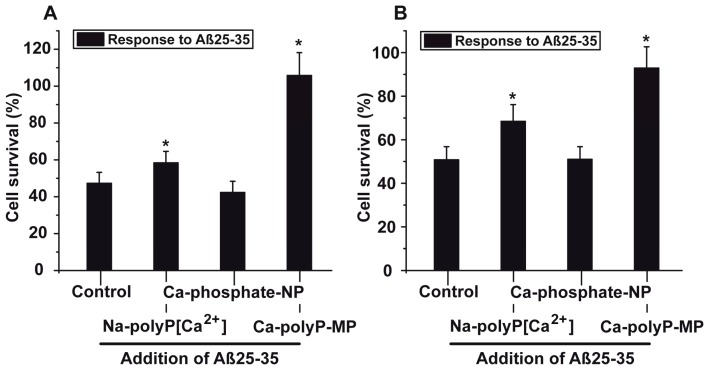
Protective effect of polyP against the toxic effect displayed by the fragment of the amyloid β-protein; the experiments were performed with (**A**) PC12 cells or with (**B**) primary rat cortex neurons. The cells remained either untreated, or were pre-incubated with 30 μg/mL of the phosphate/polyP samples (either Na-polyP[Ca^2+^], Ca-phosphate-NP, or Ca-polyP-MP) for 24 h. Then the cells, at a concentration of 8 × 10^4^ cells/mL, were incubated with the 6 h pre-incubated Aβ25-35 fragment. After a following period of 12 h the viability of the cells in the respective assays was determined with the MTT assay. From those values, the cell survival rate was calculated and the values are given in % of the respective controls (without phosphate/polyP and Aβ25-35). Data ± SD (ten independent experiments) are give; the significance (*) is calculated *p* < 0.05.

The results with PC12 cells (8 × 10^4^ cells/mL) show that in the assays with 5 μM Aβ25-35, the phosphate (Ca-phosphate-NP) pre-treated cells did not show any significant increase in cell survival compared to the control. However, the cells that had been pre-incubated with Na-polyP[Ca^2+^] or Ca-polyP-MP show a significant resistance against the toxic effect of Aβ25-35 (Figure 5A). Striking is the effect of Ca-polyP-MP on cells during the pre-incubation period. Those cells reached the same survival rate, when compared to cells that are not incubated with the fragment Aβ25-35.

**Figure 6:**
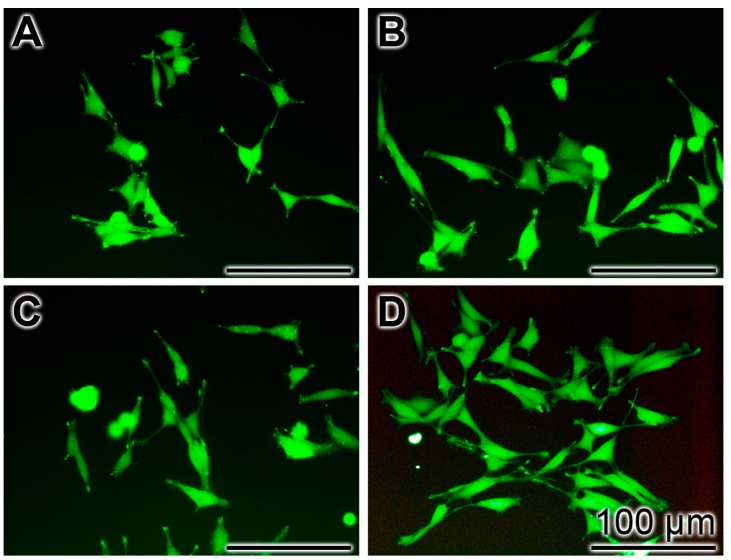
Frequency of cells in a microscopic view-frame pre-treated with phosphate/polyP (24 h) and treated with Aβ25-35 (6 h). The PC12 cell cultures remained untreated (**A**) or were pre-treated with 30 μg/mL of Na-polyP[Ca^2+^] (**B**); Ca-phosphate-NP (**C**); or Ca-polyP-MP (**D**). After staining with Calcein AM the cells were inspected by fluorescence microscopy.

In order to support and express the cytoprotective effect of the Ca-polyP-MP towards the peptide the PC12 cells remained either untreated or were pre-treated with 30 μg/mL of Na-polyP[Ca^2+^], Ca-phosphate-NP or Ca-polyP-MP (24 h) Then, the cells were treated with 5 μM Aβ25-35 for 6 h followed by staining with Calcein AM. The fluorescence images from the untreated (Figure 6A) and Ca-phosphate-NP-treated cells (Figure 6C) exhibit only relatively few cells, while the cultures treated with Na-polyP[Ca^2+^] (Figure 6B) and especially those incubated with Ca-polyP-MP (Figure 6D) display markedly more cells in the microscopic view-frame.

The results of these experiments were corroborated with primary rat cortex neurons (Figure 5B). Also those neurons (8 × 10^4^ cells/mL), pre-incubated with 30 μg/mL of Na-polyP[Ca^2+^] or Ca-polyP-MP for 24 h, showed a significant higher survival rate after exposure to Aβ25-35, when compared to the non-treated controls (not pre-treated). While cells, which remained untreated or had been pre-treated with 30 μg/mL of Ca-phosphate-NP showed a survival rate towards the toxic effect of Aβ25-35 of only ~50%, this value increased to 69% (for Na-polyP[Ca^2+^]) or even 92% (Ca-polyP-MP).

In preliminary studies we found that a co-addition of phosphate nanoparticles to the Aβ25-35 only marginally, and not significantly, elicit a cytoprotective effect seen for the polyP nanoparticles, if added during a pre-incubation period (to be published).

Furthermore, it is well established that among the different peptides of the amyloid β-protein, besides of the Aβ25-35 peptide, also the Aβ1-42 form is toxic to neuronal cells in vitro [29,30]. Exposure primary rat cortex neurons with 5 μM of Aβ1-42 for 24 h resulted in a strong reduction of the number of viable cells to 35.7%, as compared to the untreated controls. However, if neurons that had been pre-incubated for 24 h with 30 μg/mL of Na-polyP[Ca^2+^], were applied and exposed to Aβ1-42 the number of viable cells increased to 82.7% (when compared to the controls).

### 2.6. Modulation of the Intracellular ATP Pool in Cells in the Absence or Presence of Aβ25-35 and Phosphate/polyP

**Figure 7:**
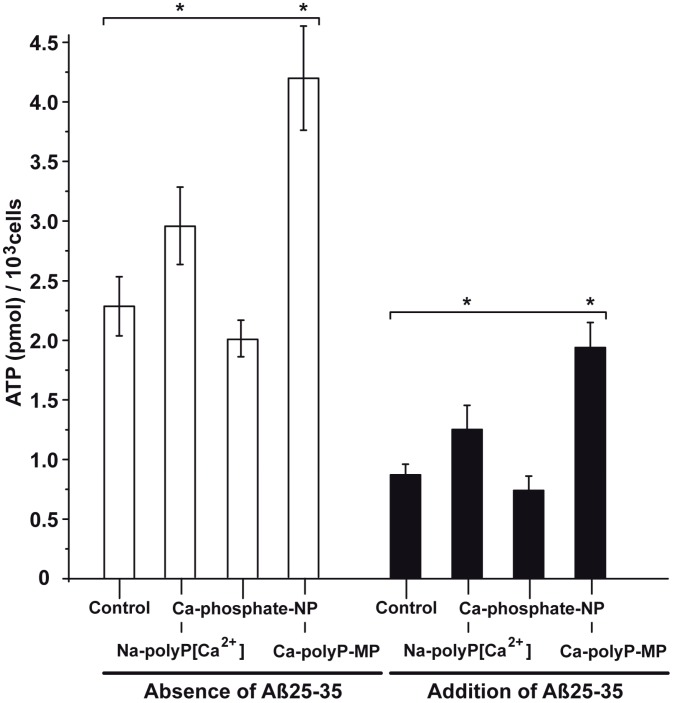
Change of the intracellular ATP pool in PC12 cells in dependence on the exposure to phosphate/polyP and/or Aβ25-35. In the first series the cells were exposed only to phosphate/polyP and not to Aβ25-35; open bar. In the Aβ25-35 test series (closed bars) the cells were treated with 5 μM of the peptide and remained either untreated (control) or were exposed to 30 μg/mL of Na-polyP[Ca^2+^], Ca-phosphate-NP, or Ca-polyP-MP, as indicated. The values came from five parallel assays; the means as well as the standard deviations are given. The significant differences between the values (control [minus phosphate/polyP]) and the test samples (Na-polyP[Ca^2+^] and Ca-polyP-MP) are indicated (*) *p* < 0.01.

The PC12 cells (8 × 10^4^ cells/mL) were incubated in the absence or presence of Aβ25-35 and then used for the determination of the intracellular ATP pool. In the absence of the peptide and also the absence of the phosphate samples (control), the ATP measures 2.27 ± 0.28 pmol of (intracellular) ATP (10^3^ cells). The addition of Na-polyP[Ca^2+^] significantly increased the level by 34%, while Ca-phosphate-NP caused no effect (an insignificant drop by 6%). However, if the cells were supplemented with Ca-polyP-MP, a significant increase of the pool by 91% is measured (Figure 7).

If the cells were incubated for 24 h in the presence of 5 μM Aβ25-35 the ATP pool dropped to 0.87 ± 0.09 pmol (10^3^ cells). If the cultures were treated together with the peptide and 30 μg/mL of Ca-phosphate-NP no significant change was measured. However, if they were treated with polyP and together with the peptide a significant increase was seen; with Na-polyP[Ca^2+^] the level increases to 1.26 ± 0.02 pmol (10^3^ cells), and with Ca-polyP-MP to 1.94 ± 0.21 pmol (10^3^ cells); Figure 7.

## 3. Discussion

One of the hallmarks of AD is the presence of two specific structures in the brain the senile plaques and the neurofibrillary tangles, which are correlated and accompanied with neuron death [31]. While Aβ25-35 is one main component of the senile plaques it is the Tau protein, in its phosphorylated form, which is the main component of the neurofibrillary tangles [32]. In the extracellular space, an abnormal deposition of wrongly processed and aggregated amyloid β-protein/peptides and Tau proteins occurs [33]. Recent studies revealed that during AD pathogenesis misformation/misfolding of Tau protein proceeds downstream of amyloid β-protein accumulation [34]. The misfolded amyloid β-protein/peptides has been implicated as a major component involved in initiation of apoptotic neuronal cell death [21,35,36]. In turn, efforts have been undertaken to develop new potential therapeutics that prevent neuronal death through the prevention of misfolding of amyloid β-protein and Tau, as well as of the subsequent apoptotic process [37]. Flupirtine [38] and memantine [8] have been elaborated by us as modulators of the NMDA receptor and consequently as neuroprotective agents, a finding that has later been confirmed by others [39,40]. Memantine has even been proposed to be applied in human as both mild and moderate-to-severe AD.

It has been demonstrated that heat shock protein(s) can reduce the neurotoxic activity of Tau [41] and amyloid β-protein/peptides [42]. Those heat shock protein(s) require energy for the folding process, usually in form of ATP [43]. They function not only intracellularly, but also extracellularly [44]. ATP is surely present also in the extracellular space but there it is prone to hydrolytic dephosphorylation through the enzyme alkaline phosphatase (ALP; [45]). The extracellular polyP has recently been proposed to act as “metabolic fuel” [15,16,46], and could provide a further depot for biochemically useful energy. This inorganic polymer has also been identified in the central nervous system at concentrations between 25 and 120 μM (~5 μg/mL, with respect to phosphate) [13,47]. Since for many years, polyP has been proven to function as a modulator of the transient receptor potential cation channel A/1 (TRPA1) and of the transient receptor potential cation channel subfamily M/8 (TRPM8), it has been suggested that polyP is involved in neuronal signaling [14]. Furthermore, experiments suggested that short and medium size polyP (chain length up to 100 phosphate units) does not activate apoptotic cascade in neurons and astrocytes [48]. Soluble polyP has been suggested to interact with the P2Y purinoceptor 1 through which it leads to an activation of the phospholipase C, resulting in a release of inositol 3 phosphate and an elevation of Ca^2+^ level in the cytosol [48]. On the other side, the Ca-polyP nanoparticles/microparticles are most likely taken up by the cells via endocytosis [16].

It is well established that mitochondrial dysfunction in AD is associated not only with enhanced oxidative stress, but also with reduced ATP generation [49]. Based on our resent finding showing that amorphous Ca-polyP induces ATP synthesis in bone-like cells [46], we raised now the question if brain-like cells, PC12 cells neuron-related cells and/or primary rat cortex neurons, can be protected against the toxic effect elicited by Aβ25-35 through the upregulation of the ATP pool. In our approach, we applied three phosphate formulations, Na-polyP, complexed to Ca^2+^ (Na-polyP[Ca^2+^]), orthophosphate nanoparticles (Ca-phosphate-NP), and finally Ca-polyP microparticles (Ca-polyP-MP). While Na-polyP[Ca^2+^] is highly soluble and acts by binding to a cell-surface receptor, the two particle preparations, Ca-phosphate-NP and Ca-polyP-MP, are less soluble. Ca-polyP-MP will be taken up into the cells by endocytosis [15,16]. It is important to stress that, under the conditions applied, the orthophosphate particles are crystalline, while the polyP particles are amorphous. In general, minerals in the amorphous state are functionally more active that crystalline mineral deposits, due to their higher solubility, and in turn their more efficient signaling potential [50,51,52]. The applied Ca-polyP-MP, similar to those that are physiologically present in the acidocalcisomes [14], are also prone to hydrolytic attack to the intra- as well as the extracellular ALP [46,53]. It should be mentioned that Ca-polyP has lost its Na^+^-chelating propensity and consequently the adverse functions of this polymers on cells [54].

The three phosphate formulations, at the concentration used (30 μg/mL), did not cause any significant effect on viability of PC12 cells. If the cells were exposed to the Aβ25-35 fragment an about 50% reduction of cell survival was measured after 6 h incubation. This Aβ25-35-mediated cell toxicity was significantly reduced by Na-polyP[Ca^2+^] by 20% if the polymer was added to the cells 24 h prior to the addition of the fragment of the amyloid β-protein. No toxic effect was measured if the cells were exposed to Aβ25-35 after pre-incubation with Ca-polyP-MP for 24 h. The cytoprotective effect of Ca-polyP-MP against Aβ25-35 exposure was confirmed with primary embryonic rat cortex neurons. Furthermore, we used also the Aβ peptide Aβ1-42 and confirmed the effects, seen with Aβ25-35. The degree of toxicity displayed by Aβ25-35 was dependent on the time of pre-incubation in aqueous solution, as described before [21,22]. The maximum of toxicity was reached already during the first 6 h; longer pre-incubation phases did not change the degree of neurotoxicity. During this time of dissolution, the fragment of the amyloid β-protein underwent spontaneous aggregation [55].

In a preceding study, it has been reported that exposure of mammalian cells, in that study SaOS-2 cells have been used [46], to amorphous Ca^2+^-polyP nanoparticles with a globular size of ~100 nm causes a ~2.5-fold increase of the intracellular ATP level, and an even ~10-fold rise in the extracellular ATP concentration. The determinations, presented here, show that PC12 cells respond to a 24 h-incubation with Ca-polyP-MP with a doubling of the intracellular ATP pool. All of the other phosphate formulations did not cause any significant alteration.

The reaction/signal transduction chain through which polyP, if extracellularly applied, changes the intracellular metabolism, is not yet known. From studies with isolated mitochondria, it is known that the permeability of mitochondrial membranes is altered in response to polyP [56]. At physiological concentrations, polyP can regulate calcium concentration by mitochondrial permeability transition pore opening, suggesting a potential regulatory role during apoptosis. As mentioned above, polyP is assumed to bind to P2Y purinoceptor 1, resulting in a modulation of the intracellular Ca^2+^ level. In our previous studies, we found that microparticles prepared from Ca-polyP are most likely taken up by the cells via endocytosis [16,46]. In addition, unpublished studies revealed that the polyP microparticles undergo an intracellular sequential disintegration [16,57]. During this process, polyP is surely released and undergoes enzymatic cleavage by the intracellular ALP. In analogy with the recent findings that extracellular polyP is prone to hydrolytic degradation by the ALP [11], resulting in an upregulation of the intracellular, as well as the extracellular ATP [46,58], we propose that also intracellularly, this inorganic polymer can function in the same reaction chain and transfers the metabolic energy stored in its energy-rich phosphoanhydride bonds to adenosine monophosphate (AMP) or ADP; the additional enzyme required for this process, the adenylate kinase, also exists in this compartment [59].

## 4. Materials and Methods

### 4.1. Materials

Sodium polyphosphate (Na-polyP of an average chain of 40 phosphate units) was obtained from Chemische Fabrik Budenheim (Budenheim; Germany).

### 4.2. Phosphate/Polyphosphate Sample Fabrication

The polyP particles were prepared as described before [15]. In brief, Na-polyP (2 g) was dissolved in 100 mL of distilled water; the resulting pH value was increased from pH 3.4 to 10 with 2 N NaOH. Then 60 mL of a CaCl_2_ solution (5.6 g CaCl_2_·2H_2_O; #C3306 Sigma, Taufkirchen; München, Germany) were added dropwise to the polyP solution. After stirring for 12 h the particles formed were collected by filtration through Nalgene Filter Units (pore size 0.45 μm; Cole-Parmer, Kehl/Rhein; Wertheim-Mondfel, Germany). Then the particles were washed twice with ethanol to remove the unbound Ca^2+^. Finally, the amorphous microparticles, “Ca-polyP-MP”, were dried at 60 °C overnight.

The particles formed from sodium orthophosphate (tri-sodium phosphate; Sigma #342483) were prepared in the same way. Two g of tri-sodium phosphate were dissolved in 100 mL of water; the pH value was adjusted to pH 10; then 60 mL of the CaCl_2_ solution (containing 5 g CaCl_2_) were added. The resulting crystalline particles, Ca-phosphate-NP, were processed as described above.

### 4.3. Fourier Transformed Infrared Spectroscopy and X-ray Diffraction 

Fourier transformed infrared (FTIR) spectroscopy was performed with a Varian 660-IR spectrometer with Golden Gate ATR auxiliary (Agilent, Darmstadt, Germany). X-ray diffraction (XRD) of the dried powder samples was conducted in a Philips PW1820 diffractometer with monochromatic Cu-Kα radiation (λ = 1.5418 Å, 40 kV, 30 mA, 5 s, Δ*θ* = 0.02) [60].

### 4.4. Microscopy

Scanning electron microscopy (SEM) was conducted with a HITACHI SU8000 electron microscope (Hitachi High-Technologies Europe, Krefeld, Germany). Light microscopy was performed with a VHX-600 Digital Microscope from KEYENCE (Neu-Isenburg, Germany).

### 4.5. PC12 Cells

Rat pheochromocytoma cells (PC12 cells) were obtained from Sigma (#88022401) and cultivated in RPMI 1640 medium (Sigma) and heat-inactivated horse serum (10%)/heat-inactivated fetal bovine serum (5%), together with gentamicin; incubation was performed in a humidified atmosphere of air (95%) and CO_2_ (5%). The cells were plated at a density 4 × 10^3^ cells in 96-well (working volume 200 μL)- or 4 × 10^4^ cells in 6-well (2 mL) plate for the indicated periods of time.

Three different phosphate preparations were added to the culture: (i) Na-polyP complexed in a stoichiometric ratio to Ca^2+^ (molar ratio of 2 (with respect to the phosphate monomer); abbreviated as Na-polyP[Ca^2+^]), as described in [54]; (ii) Ca-phosphate-NP; and, (iii) Ca-polyP-MP (40 phosphoanhydride bond-linked phosphate units). The concentrations are given with the respective experiments and were usually 30 μg/mL.

For microscopic visualization the cells were stained with 5 μM Calcein AM (Sigma). Then they were inspected by fluorescence microcopy at an excitation wavelength of 494 nm and an emission of 540 nm.

### 4.6. Cell Viability Assays

The toxicity of the phosphate preparations was determined after an incubation period of the PC12 cells in medium/serum for 72 h. The cells were seeded into 48-well plates at an initial density 2 × 10^4^ cells/mL. Then, the viability of the cells was determined with the 3-[4,5-dimethyl thiazole-2-yl]-2,5-diphenyl tetrazolium (MTT; #M2128, Sigma) assay. The cells were detached (by carefully pipetting up and down with a 10 mL pipette) and a 2 mL cell suspension was aspirated and subsequently incubated with fresh medium containing 200 μL of MTT for 4 h in the dark. Subsequently, the remaining MTT dye was removed and 200 μL of dimethyl sulfoxide (DMSO) was added to solubilize the formazan crystals. Finally, the optical densities (OD) at 570 nm were measured using an enzyme-linked immunosorbent assay (ELISA) reader/spectrophotometer [61]. Cell viability was expressed as a percentage of the untreated control (without phosphate/polyP). Ten parallel experiments were performed.

### 4.7. Aβ-Induced Cell Toxicity

Previously, we found that the Aβ25-35 sample, should be pre-incubated in a stock solution (900 μM in distilled water) for five days to develop the full toxicity [21]. In the present study, we performed a time kinetics to assess the toxicity elicited by Aβ25-35 (#A4559, Sigma) by pre-incubating the Aβ25-35 stock solution in distilled water for a period between 6 and 24 h in the absence or presence of polyP. The polymer, Na-polyP[Ca^2+^], was added at a concentration of 30 μg/mL. Subsequently, the corresponding volume of the sample was taken and added to PC12 cells (2 × 10^4^ cells/mL) to reach a final concentration of 5 μM in the cell assays. The cells were incubated for 12 h. After termination, the viability of the cells in the culture was determined by the MTT assay (see above). From those results, the cell survival rate in % of the controls (without Aβ25-35), was calculated.

For testing the toxicity of the Aβ25-35 peptide, the stock solution was diluted to a final concentration in the toxicity assays of 5 μM [21,22]. Prior to the addition of Aβ25-35 the cells were pre-incubated for 24 h in medium/serum at a final concentrations of 30 μg/mL with Na-polyP[Ca^2+^], Ca-phosphate-NP or Ca-polyP-MP; the controls were incubated in parallel and did not contain any phosphate sample. Then, the medium was removed and the Aβ25-35 sample was added. Incubation was performed for 12 h; finally, the viability of the cells was determined with MTT. Cell viability is given in percent of the untreated control (without Aβ25-35 and without phosphate/polyP).

In one series of experiments, the Aβ1-42 fragment (#A9810, Sigma) had been used in parallel to the toxicity studies. Again, a stock solution (900 μM in distilled water) had been prepared. After keeping for 24 h, aliquots had been taken and added to primary rat cortex neurons to reach a final concentration of 5 μM. After incubation for 24 h, in the absence or presence of 30 μg/mL Na-polyP[Ca^2+^] the cell number was determined.

### 4.8. Primary Culture of Cortical Neurons

Primary rat cortex neurons isolated from 18-day old rat embryos were obtained from GIBCO/Thermo Fisher Scientific (Langenselbold, Wiesbaden, Germany). They were cultivated in Neurobasal Medium (from GIBCO/Thermo Fisher Scientific), as described in [38,62]. Five days old cultures were used for the studies. The experiments were performed in 96-well/or 6-well plates.

The assay regimen with Aβ25-35 as well as the pre-incubation protocol of the cells with the phosphate samples was the same as for PC12 cells.

Cell toxicity was determined after 12 h with the MTT assay [63].

### 4.9. Determination of the ATP Level in PC12 Cells

PC12 cells were cultivated in 12-well plates until they reached about 75% of confluency (8 × 10^4^ cells/mL). Then, the cells were continued to be incubated for 24 h in the absence of any polyP, or the presence of the three phosphate samples in the absence of presence of Aβ25-35. Finally, ATP was extracted from the cells [64,65,66], and the ATP concentration was determined by using the ATP luminescence kit (NO. LL-100-1, Kinshiro, Toyo Ink; Fukuoka, Japan), as described in [46]. After establishing a standard curve for given ATP concentrations, the absolute amount of ATP was extrapolated and is given as pmol/10^3^ viable cells. In parallel assays, the number of viable cells were determined by the MTT cell viability assay [67].

Prior to the ATP determination the cells were pre-incubated for 24 h with 30 μg/mL of Na-polyP[Ca^2+^], Ca-phosphate-NP or Ca-polyP-MP; again, the controls were incubated in parallel and they did not contain any phosphate sample.

### 4.10. Statistical Analysis

After verifying that the respective values follow a standard normal Gaussian distribution and that the variances of the respective groups are equal, the results were statistically evaluated using the independent two-sample Student’s *t*-test [68].

## 5. Conclusions

**Figure 8:**
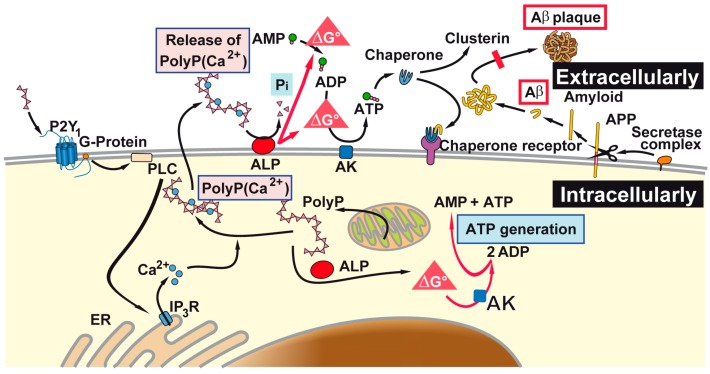
As outlined, polyP microparticles that can be taken up by the cells, or formed there intracellularly, are prone to the alkaline phosphatase (ALP). In analogy to the acidocalcisomes, polyP microparticles are also formed intracellularly, and can subsequently be released by the cells. It is sketched that the (proposed) pathways of ATP generate both intra- and extracellularly Gibbs free energy that is released during the hydrolysis of the anhydride bond in polyP into both compartments. This process can occur both extracellularly [58] and intracellularly. Those adenosine nucleotides provide the energy for the extracellular chaperones which might prevent misfolding of amyloid plaques (abbreviated as Aβ). AK: adenylate kinase; AMP: adenosine monophosphate; ADP: adenosine diphosphate; ATP: adenosine triphosphate; ER: endoplasmic reticulum.

The described experiments in the present study indicate that the physiologically occurring polyP microparticles/nanoparticles, prepared from Ca-polyP, can effectively block the toxic effect elicited by the toxic peptide fragment Aβ25-35; see scheme in Figure 8. This inhibition is attributed to an elevation of intracellular ATP level, which has been described to be one factor for neuronal cell death in AD, or in AD-model systems. Surely further studies are required to elucidate if the observed effect of the polyP microparticles/nanoparticles to increase the ATP level intracellularly and can also be extended to the extracellular ATP pool [58]. Also, in this extracellular compartment ATP, or equivalent metabolic energy, is required for the heat shock protein-mediated folding of proteins in general and of the amyloid precursor protein and/or it derivatives [69]. There, in the extracellular space, ADP (or perhaps also ATP) could feed, e.g., the chaperone clusterin with its ADP binding motif [70], with energy prevent misfolding of the amyloid precursor protein. Intracellularly, the polyP microparticles are enzymatically hydrolyzed via the ALP.

The data reported in this contribution might help to develop new routes for a therapeutic intervention in AD pathophysiology. Methods have been worked out to deliver drugs, encapsulated into nanoparticles/microparticles, which can cross the blood-brain barrier [71,72]. Through this route, the delivery of the potential polyP compound across the blood-brain barrier appears to be promising, since this structural barrier undergoes dysfunction during the AD progression, facilitating particle transport [73,74]. This conclusion is also strengthened by a previous report [17], showing that polyP is not only stabilizing the structure of the amyloid β-protein but also might have a beneficial effect on the progression of the Alzheimer’s disease in patients. However, future in vitro studies must be performed to determine, if cells pretreated with the peptide of the amyloid β-protein and are in the process to undergo apoptosis can be rescued by soluble or particulate polyP. In case such a rescuing effect cannot be found, polyP still remains a candidate molecule for prevention. But even then, polyP might be possible to lower the amyloid β-protein/fragments load in patients; recent studies suggest that peptides of the amyloid β-protein act as seeding molecules and start an amyloid β-protein seeding cascade [75].

## Abbreviations

AD	Alzheimer’s disease
Aβ	Amyloid β-peptides
Aβ25-35	amyloid β-protein fragment 25-35
ADP	adenosine diphosphate
AMP	adenosine monophosphate
ATP	adenosine triphosphate
Ca-polyP-MP	Ca-polyP microparticles
DMSO	dimethyl sulfoxide
ELISA	enzyme-linked immunosorbent assay
MTT	3-[4,5-dimethyl thiazole-2S-yl]-2,5-diphenyl tetrazolium
Na-polyP	Sodium polyphosphate
Na-polyP[Ca^2+^]	Na^+^ salt of polyP complexed in a stoichiometric ratio to Ca^2+^
PC12 cells	Pheochromocytoma cells
polyP	Polyphosphate
SEM	Scanning electron microscopy
TRPA1	Transient receptor potential cation channel A/1
TRPM8	Cation channel subfamily M/8
